# ENGAGING YOUTH AND HEALTH CARE PROFESSIONALS: DEVELOPING THE YCARE PROGRAM FOR YOUNG CARERS

**DOI:** 10.1093/geroni/igae098.0966

**Published:** 2024-12-31

**Authors:** Melinda Kavanaugh

**Affiliations:** University of Wisconsin Milwaukee, Milwaukee, Wisconsin, United States

## Abstract

Children and youth ages 8-19 who provide care, “young carers,” are tasked with the same caregiving as adults, including bathing, feeding, toileting, and managing complex assistive devices. Yet, young carers receive little to no caregiving guidance or training in community or health care settings, leaving them isolated not only from peers but from health care professionals. Indeed, over 70% of young caregivers for people living with complex neurological disorders (Huntington’s disease and ALS) reported no education or training in care provision, leaving them to “wing it”, or rely on “common sense,” while stating they have few peers or professionals with whom they can share experiences and build support. Health care professionals who provide guidance and training to adult caregivers, also have little understanding of young carers, and how best to engage and support them. The lack of connections between young carers and health care professionals limits the opportunity to build support and trust between young carers and the health care system. This presentation details the iterative, multidisciplinary development of the YCare program, a peer-engaged, training, skill, and support program for young carers. We will also discuss how multidisciplinary health care professionals, including OT, PT, Social work, respiratory and speech therapies were recruited and trained to deliver the YCare program - connected young carers and much needed care skills and support.

